# Evaluating behavior change factors over time for a simple vs. complex health behavior

**DOI:** 10.3389/fpsyg.2022.962150

**Published:** 2022-09-08

**Authors:** L. Alison Phillips, Kimberly R. More

**Affiliations:** ^1^Department of Psychology, Iowa State University, Ames, IA, United States; ^2^Department of Psychology, School of Humanities, Social Sciences and Law, University of Dundee, Dundee, United Kingdom

**Keywords:** behavioral maintenance, theories of behavior change, exercise, adherence, habit formation

## Abstract

**Background:**

Researchers are working to identify dynamic factors involved in the shift from behavioral initiation to maintenance—factors which may depend on behavioral complexity. We test hypotheses regarding changes in factors involved in behavioral initiation and maintenance and their relationships to behavioral frequency over time, for a simple (taking a supplement) vs. complex (exercise) behavior.

**Methods:**

Data are secondary analyses from a larger RCT, in which young adult women, new to both behaviors, were randomly assigned to take daily calcium (*N* = 161) or to go for a daily, brisk walk (*N* = 171), for 4-weeks. Factors (intentions, self-efficacy, intrinsic motivation, self-identity, habit strength) were measured weekly. Multi-level modeling evaluated their change over time. Bivariate correlations and multiple regression determined the relationships between factors and the subsequent-week behavioral frequency (self-report and objective).

**Finding:**

Results were partly in-line with expectations, in that individuals’ intentions and self-efficacy predicted initial behavioral engagement for both behaviors, and habit strength increased for both behaviors, becoming a significant predictor of behavioral frequency in later weeks of the study in some analyses. However, results depended on whether the outcome was self-reported or objectively measured and whether analyses were bivariate or multivariate (regression).

**Discussion:**

The factors theorized to play a role in behavioral maintenance (intrinsic motivation, self-identity, and habit strength) started to develop, but only habit strength predicted behavioral frequency by study-end, for both behaviors. Differences in initiation and maintenance between behaviors of differing complexity may not be as stark as theorized, but longer follow-up times are required to evaluate maintenance factors.

## Introduction

Far too few individuals meet recommendations for engagement in healthy behaviors, including sufficient regular exercise (<5% of the US population), healthy diet (<25%), and medication adherence (<50%) (Spring et al., 2012). Interventions are needed to improve our success at both initiating and maintaining health behavior change (Sheeran et al., 2017). To improve the success of behavior change interventions, researchers are working to improve our understanding of the dynamic factors involved in behavioral initiation (including preparation for initiating the behavior, initial attempts at the behavior, and short-term repetition of the behavior; Prochaska and Velicer, 1997), behavioral maintenance (including longer-term behavioral repetition and relapse prevention; see Dunton et al., 2022), and the dynamic transitions between these phases. Ongoing research questions that we address in the present study include: first, what factors promote behavioral initiation and which become more or less important as behavior is repeated, heading toward behavioral maintenance? And second, how might these factors and their relationship to behavioral engagement (i.e., frequency) over time depend on the complexity of the behavior—how many steps and how much time is required to prepare for and enact the behavior and whether the behavior as meaningfully separable parts that can be differentially targeted with intervention (see Phillips and Mullan, 2022)?

Widely studied theoretical factors involved in behavioral initiation include self-efficacy (Health Action Process Approach; Schwarzer, 2008), motivation that varies in quality from intrinsic to purely extrinsic (Self-Determination Theory; Deci and Ryan, 2000), and behavioral intentions (Theory of Planned Behavior; Ajzen, 1991), among others. These factors are posited to differ from factors involved in behavioral maintenance (Rothman, 2000), and recent research has found that they are better predictors of behavioral engagement among those in an initiation vs. maintenance stage of change (More and Phillips, 2022).

Factors posited to be involved in behavioral maintenance include behavioral habit, autonomous motivation, and self-identity. The factors are included in some theories and frameworks presented in existing literature, such as the multi-process action control approach (M-PAC; Rhodes et al., 2021), habit theory (e.g., Lally and Gardner, 2013; Gardner, 2015), and in a review of 117 behavioral theories regarding processes of behavioral maintenance by Kwasnicka et al. (2016).

Probably the most widely studied behavioral maintenance factor has been behavioral habit strength. Habits can be defined as automatic impulses to engage in a behavior that are triggered by learned/conditioned context cues (see Gardner, 2015), or as an automatic association between a context and an action that is formed through repeated and rewarded action in that context (see Wood et al., 2022). Automaticity, and therefore habit strength, is considered a continuous variable that varies in type (e.g., non-conscious vs. efficient; Moors and De Houwer, 2006). Habit strength is associated with frequency and consistency of behavioral engagement over time, including in the face of common barriers such as time restraints and stress (Verplanken, 2006; Wood and Neal, 2007; Gardner, 2015). For complex behaviors in particular, researchers have posited that intrinsic motivation is a critical component of habit development and continued habitual action of health-promotive behaviors (Phillips et al., 2016; Phillips, 2020; Phillips and Mullan, 2022)^1^. There is observational evidence that autonomous motivation facilitates habit formation and maintenance of complex health behaviors, such as exercise (Gardner and Lally, 2013; Phillips et al., 2016). Gardner and Lally (2013) found that behavioral repetition was associated with habit formation (stronger habits) only when behavior was autonomously motivated. Phillips et al. (2016) found that intrinsic rewards (e.g., enjoyment of and stress reduction from exercise) predicted greater exercise frequency through stronger habits (vs. intentions) for those in a maintenance stage of change. Additional, theoretical arguments are presented in the literature regarding the necessity of intrinsic reward for habit development and maintenance (see Phillips et al., 2016; Phillips, 2020; Phillips and Mullan, 2022). Briefly, without a reward, any habit would cease, eventually, as has been acknowledged since the earliest habit research by behaviorists (e.g., Skinner, 1953). Given the greater time and effort required to enact complex behaviors, these would be even easier to cease if they stopped being rewarding, then simple habits which may be able to continue without conscious awareness of a reward or lack thereof (in the case of no barriers to continuing the behavior). It is important to note that there are other types of motivation that may also facilitate habit formation, although they are not the focus of the current investigation; for example, autonomous motivation includes intrinsic motivation (e.g., behavioral enjoyment) and identified regulation (motivation to behavior in line with one’s personal values).

In addition to habit and intrinsic motivation, we focus on behavioral self-identity (e.g., identity as an exerciser) as a mechanism of behavioral maintenance. Self-identity based on one’s engagement in a behavior is associated with behavioral maintenance (Caldwell et al., 2018; Verplanken and Sui, 2019) but has primarily been studied in exercise contexts (Anderson and Cychosz, 1994; Rhodes et al., 2021). Identity may play both a reflective and reflexive role in behavioral engagement (Stets and Burke, 2000; Strachen et al., 2009). Acting in line with our identity is rewarding (causes positive affect; e.g., Stets and Burke, 2000) and may therefore serve as a form of intrinsic reward for continued behavioral engagement; it may also therefore serve a direct role in habitual action, given that intrinsic behavioral rewards strengthen habit. Identity also plays a reflective (conscious, deliberative) role in behavioral engagement, in that we behave in ways that we reflect are in line with our identities (Burke, 2006).

Although some behavior theories propose transitions in predictive factors from initiation through maintenance, little research has empirically evaluated how these factors change over time and how they change in relative importance for promoting behavioral engagement over time, with behavioral repetition. Additionally, researchers have yet to evaluate how behavioral initiation and maintenance factors and the transition between them for predicting behavioral engagement may differ across behaviors that vary in complexity. For example, a simple behavior, such as taking pill, may be motivated by the desire for better health (identified regulation; Deci and Ryan, 2000), but not necessarily intrinsic motivation, which is posited to be more important for initiation (and maintenance) of complex behaviors, such as exercise (Phillips et al., 2016). Further, strong habit may be sufficient for maintenance of a simple behavior, in a stable context, whereas a complex behavior may require self-identification with the behavior and intrinsic motivation, in addition to or as part of a strong habit (Phillips and Mullan, 2022).

Therefore, the purpose of the current study is to evaluate changes in behavior change factors over time and their importance for predicting subsequent behavior for a simple vs. a complex health behavior. Calcium supplementation and exercise were chosen for the current study for the following reasons: First, they fit with the concept of relatively simple and complex behaviors, respectively, since taking a pill takes relatively fewer steps and less time to prepare for and enact than does going for a brisk walk (exercising) (see Phillips and Mullan, 2022). Second, these behaviors are both target health behaviors for disease prevention and management and therefore have clinical importance. Third, existing research has measured/observed and targeted via intervention medication-taking and exercise-related habits (e.g., Phillips et al., 2016). Lastly, these behaviors can be objectively assessed via sensors in addition to self-reported measures.

Based on widely used theories and frameworks, we focus the current investigation on the behavioral initiation factors of self-efficacy for engaging in the behavior (perceived behavioral control, task self-efficacy), behavioral intentions, and intrinsic motivation; and on proposed behavioral maintenance factors—habit strength, identity, and intrinsic motivation.

We test the following hypotheses: (1) behavioral intentions and self-efficacy (behavioral initiation factors) will be high and remain high or decrease over time for individuals newly engaging in a simple (taking a daily calcium supplement) or complex (going for a daily, brisk, 20 + minute walk) behavior; (2) intrinsic motivation (initiation and maintenance factor for complex behaviors) will start and remain low for calcium supplementation but will increase over time for exercise; (3) habit strength (a maintenance factor) will start low and increase over time for both behaviors; (4) behavioral identity (a maintenance factor) will start and remain low for calcium consumption and will start low but increase over time for exercise.

Additionally, regarding their role in behavioral prediction, we hypothesize that (5) behavioral intentions and self-efficacy will predict initial adherence to calcium supplementation (self-reported and objectively measured, weeks 1 and 2); whereas habit strength will predict calcium supplementation adherence in later weeks (weeks 3 and 4); and (6) behavioral intentions, self-efficacy, and intrinsic motivation will predict exercise frequency and step-counts in initial weeks (weeks 1 and 2); whereas habit strength, intrinsic motivation, and exercise identity will predict exercise frequency and step-counts in later weeks (weeks 3 and 4).

## Materials and methods

### Participants and procedure

Young adult women study volunteers, who did not previously take calcium supplements and got less than recommended amounts of physical activity (self-reported getting less than 150 min of moderate intensity or greater activity per week), were randomly assigned to have a target behavior of taking a calcium supplement (*N* = 185) or to go for a brisk, 20-min, walk (*N* = 182), on at least 5 of 7 days of each week, for 4 weeks. In the whole sample, approximately 76.6% identified as White, 4.6% as Black, 3.8% as South Asian, 4.9% as East Asian, <1% as Native American, 7.1% as “Other,” and 2.7% chose not to respond regarding their racial identity. A majority (90.5%) identified as Non-Hispanic, 8.4% identified as Hispanic, and 1.1% chose not to respond to the item regarding Ethnic identity. The average age of participants was 19.11 (SD = 1.56).

The current analyses utilize data from an intervention study that tested a habit formation strategy (action planning) with various timing-manipulations (morning vs. evening cues) and a control group. Both behaviors had the same manipulations/groups, and the data for the current analyses collapse across the experimental conditions for each behavior; however, analyses control for experimental condition. Most of the variables included in the current analyses (intentions, self-efficacy, intrinsic motivation, and identity) are not included in the main evaluation of the intervention. The main intervention study evaluates which group(s) had greater habit formation and behavioral frequency over the 4 weeks of the study (Phillips et al., in preparation). The hypotheses tested in the current analyses and in the main intervention analyses are entirely different from each other. The data took four semesters (2 academic school years) to collect, prior to the COVID-19 pandemic. Informed consent to participate in the study was provided by all participants, prior to the beginning of the baseline session, and all procedures were approved by the local institutional review board.^2^

All predictors (behavior-specific intentions, self-efficacy, intrinsic motivation, self-identity, and habit strength) were measured at baseline (in-person survey), weekly for 3 weeks (online surveys), and at a final 4-week follow-up (in-person survey). Participants were also provided with a digital sensor for their assigned behavior—a Medication Event Monitoring System Cap (MEMS Cap; Aardex, Belgium) for those taking calcium supplements, and an accelerometer (Fitbit Zip) for those in the exercise conditions. The Fitbits were covered so that participants were not able to see how many steps they were taking. The MEMS Caps gave no information regarding the participants’ behavior to the participants themselves, but recorded the exact time the bottle was opened (and a pill taken, theoretically).

Power calculations to determine sufficient sample size were conducted for the main study, to detect differences in behavior change between experimental conditions, rather than for the present analyses. We therefore conducted *post hoc* power analyses at alpha = 0.05, and observed power was 100% for all ANCOVA (i.e., to detect significant main and interaction effects with the three dummy coded co-variates for intervention condition and time and behavior as factors) and linear regression (i.e., to detect a significant increase in R2 with addition of four predictor variables—intention-efficacy, intrinsic motivation, habit strength, and identity) analyses (evaluated with SPSS and G*Power, respectively; Erdfelder et al., 1996).

### Measures

#### Intentions and self-efficacy

**FIGURE 1:**
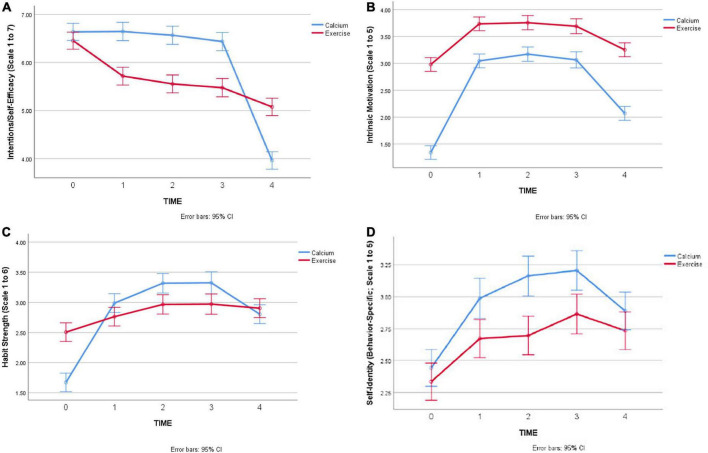
**(A)** Change in intentions-efficacy over time, by behavior. **(B)** Changes in intrinsic motivation over time, by behavior. **(C)** Changes in habit strength over time, by behavior. **(D)** Changes in behavioral identity over time, by behavior.

Items to assess intentions and self-efficacy for engaging in the target behaviors were created from guidelines for developing Theory of Planned Behavior questionnaires (see Fishbein and Ajzen, 2010). Participants were asked the items about their assigned behavior: “I intend to exercise (e.g., take a brisk walk) for 20 + minutes at a moderate-vigorous intensity on 5 + days during the next week” or “I intend to take calcium on 5 + days during the next week”; and for self-efficacy (perceived behavioral control): “I am confident that I can exercise (e.g., take a brisk walk) for 20 + minutes at a moderate-vigorous intensity on 5 + days during the next week” or “I am confident I can take a calcium supplement on 5 + days during the next week.” These two items were highly correlated with each other, at *r* = 0.85 in the calcium data and *r* = 0.76 in the exercise data. Further, we evaluated the trajectory of change in these variables over time separately, and the trajectories were equivalent (each individual trajectory was the same as seen for the combined variable, in Figure 1A). Lastly, the correlations between the intention and self-efficacy variables with outcomes were meaningfully equivalent (i.e., in size of effect and statistical significance level). Therefore, the items were combined (averaged) to represent participants’ intentions-efficacy for engaging in the target behavior in the subsequent week.

#### Intrinsic motivation

Intrinsic motivation to exercise was measured with two items from the intrinsic regulation subscale of the Behavioral Regulation of Exercise Questionnaire (BREQ-3; Markland and Tobin, 2004; Wilson et al., 2006). The items were tailored to refer to the past week as a reference point (vs. no time reference in the original items), and the calcium group was asked about calcium supplementation instead of exercise. The items are rated on a Likert scale from not true for me (1) to very true for me (5): “In the past week, I enjoyed going for my brisk walk (or equivalent activity)/taking my calcium supplement” and “In the past week, I got pleasure and satisfaction from taking my brisk walk (or equivalent activity)/taking my calcium supplement.”

#### Habit strength

Habit strength was measured with two items from the 4-item Self-Reported Behavioral Automaticity Index (SRBAI; Gardner et al., 2012). The items were rated on the Likert scale, strongly disagree (1) to strongly agree (5): “In the past week, going for my brisk walk (or equivalent activity)/taking my calcium supplement was something I did without thinking” and “In the past week, going for my brisk walk (or equivalent activity)/taking my calcium supplement was something I did automatically.”

#### Identity

The degree to which an individual identifies as an exerciser was assessed with one item from the Exercise Identity Scale (Anderson and Cychosz, 1994). It is rated on the Likert scale, strongly disagree (1) to strongly agree (5): “I consider myself an exerciser.” Participants in the calcium group were given an item created for the current study, as a literature search did not find any existing items related to medication-adherence identity: “I consider myself to be someone who makes an effort to get enough calcium.”

#### Behavioral engagement

The outcome measure for calcium supplementation was the number of days per week the supplement was taken, measured via electronic monitoring pill bottles and self-report. The self-report item was, “On how many days in the past week did you take calcium?” The outcome measures for brisk walking were the number of days per week with a brisk walk, measured via self-report, and the average daily number of steps per week, measured via an accelerometer (Fitbit Zip). The self-report item was, “On how many days in the past week did you exercise at a moderate to vigorous intensity (e.g., brisk walk)?”

### Analysis plan

#### Preliminary analyses

Some self-report/survey data and some sensor data were missing, at various timepoints, for some participants. The percentage of observed timepoints for all participants that had missing survey data is 6.8%, across both behaviors (125 observations out of 1,835 total observations). For sensor data, 14.2% of observations had missing data. The missing data were scattered throughout, and only two individuals showed true attrition, meaning they had baseline data and then were missing all subsequent follow-up timepoints; these two individuals were excluded from all analyses.

We evaluated univariate outliers by determining if there were scores on the variables that were ± 3 standard deviations from the averages for those variables. Multivariate outliers were determined through evaluating Mahalanobis distance values at standard protocol significance level of *p* < 0.001 (Tabachnick and Fidell, 2007). After accounting for univariate outliers, there were no multivariate outliers. There were only two individuals who were univariate outliers and they were outliers on only the Fitbit measure of average steps per day, per week, for weeks 1, 2, and 3. Their values were in the realm of possibility (human capability), and so we Winsorized their outlying values so that they were still greater than the other participants’ values but only 3 SD above the mean (instead of 6 and 8 SD above the mean).

There were 13 individuals who failed a random response check, where they were asked to indicate a specific response to a Likert scale item, embedded within other Likert scale items. We compared correlations between the current study variables with and without these random responders included, and the correlations/effects and significance values did not meaningfully change. Therefore, we analyzed tests of the hypotheses including all participants.

#### Tests of hypotheses

We conducted 2 (behavior) × 5 (time-point) ANCOVAs for each behavior change factor to evaluate Hypotheses 1–4—i.e., to see the average rate of change in the factors, by behavior, over time, controlling for the intervention conditions in dummy coded variables. For hypotheses 5 and 6, bivariate correlations and multiple regression analyses (controlling for intervention conditions) were conducted for each behavioral outcome at weeks 1, 2, 3, and 4, with predictor variables measured at the preceding time-point entered into the model. That is, baseline (W0) predictors were used to predict week 1 behavioral engagement, week 1 predictors were used to predict week 2 behavioral engagement, and so forth.

## Results

**Hypothesis 1 (behavioral intentions and self-efficacy will be high and remain high or decrease over time for simple and complex behaviors).** There were significant main effects for Time and Behavior, qualified by a significant interaction between Time and Behavior (*F* = 47.33, *p* < 0.001) in predicting intentions-efficacy over time (see Figure 1A, which shows 95% confidence intervals for mean levels of intention-efficacy). Intention-efficacy was not significantly different for calcium and exercise groups, at baseline, and both behavioral groups showed a marked decrease in intentions and self-efficacy at the final-timepoint. Exercise participants showed a more gradual decline than calcium participants, who only decreased in intentions and self-efficacy at the final timepoint.

**Hypothesis 2 (intrinsic motivation will start and remain low for calcium supplementation but will increase over time for exercise).** There were significant main effects of Time and Behavior in predicting intrinsic motivation over time, but these were qualified by a significant interaction between Time and Behavior (*F* = 23.57, *p* < 0.001). Figure 1B shows the results for intrinsic motivation, with 95% confidence intervals on the mean levels of intrinsic motivation for each behavior at each timepoint: As hypothesized, intrinsic motivation was higher for exercise than calcium at the start and throughout the study. However, intrinsic motivation did increase (and then decrease) for calcium consumption, which was not expected.

**Hypothesis 3 (habit strength will start low and increase over time for both behaviors).** There were significant main effects of Time and Behavior in predicting habit strength over time, which were qualified by a significant interaction between Time and Behavior (*F* = 19.54, *p* < 0.001). As seen in Figure 1C, there was an overall increase in habit strength, with a slight decrease at the final timepoint for calcium supplementation. We note that neither behavior shows an average level of habit strength = 4, which is a rough level associated with “having a habit,” since individuals have to agree on average with the statements that their engagement in the behavior is “automatic,” or habitual, to have a score of 4.

**Hypothesis 4 (behavioral identity will start and remain low for calcium consumption and will start low but increase over time for exercise).** Differently to the other variables, there was a non-significant interaction between Time and Behavior in predicting behavioral identity over time (*F* = 1.81, *p* = 0.12). There were significant main effects of Time (*F* = 21.25, *p* < 0.001) and Behavior (*F* = 32.85, *p* < 0.001), as shown in Figure 1D. Unexpectedly, the calcium group showed slightly higher identity scores at the end of weeks 1–3, than the exercise participants, with a slight decrease at the final timepoint. As expected, exercise participants did show a slight increase in exercise identity over the course of the study.

**Hypothesis 5 (behavioral intentions and self-efficacy will predict initial adherence to calcium supplementation, whereas habit strength will predict calcium supplementation adherence in later weeks).** Regarding initial adherence to calcium supplementation: In week 1, as hypothesized, intentions-efficacy was the only baseline-measured factor to predict self-reported and objectively measured calcium supplementation, in bivariate and regression analyses. However, by week 2, intentions-efficacy was not the only predictive factor of calcium adherence: intrinsic motivation was a significant predictor in both bivariate and regression analyses of self-reported and objectively measured calcium adherence. Habit strength predicted both of these outcomes, but only in bivariate analyses. Table 1A shows correlational analyses, and Table 2A shows regression analyses (for weeks 1 and 4 outcomes), for calcium supplementation variables.

**TABLE 1A:** Correlation results for calcium supplementation variables.

	Week 1		Week 2		Week 3		Week 4	
	Self-report	Objective	Self-report	Objective	Self-report	Objective	Self-report	Objective
1. Intentions-efficacy	0.363**	0.370**	0.492**	0.472**	0.487**	0.438**	0.675**	0.481**
2. Intrinsic motivation	0.017	0.013	0.282**	0.269**	0.229**	0.190*	0.359**	0.241**
3. Identity	0.099	0.055	0.085	0.066	0.096	0.122	0.341**	0.108
4. Habit strength	0.012	0.021	0.277**	0.252**	0.298**	0.358**	0.561**	0.296**

The predictors used to calculate each correlation were measured at the preceding timepoint to the outcome. Therefore, for W1 (week 1) Self-report and objective outcomes (calcium supplementation frequency), the predictors were measured at baseline (W0). For W2 outcomes, predictors were measured at W1, and so forth.

*P < 0.05 and **P < 0.01.

**TABLE 2A:** Regression results for calcium supplementation variables (weeks 1 and 4 outcomes only, for comparison and space reasons).

	B	SE	Beta	T	Sig.
**Predicting self-reported calcium supplementation in week 1**					
(Constant)	1.31	0.99		1.32	0.19
Intentions-efficacy, W0	0.66	0.14	0.37	4.84	0.000
Intrinsic motivation, W0	–0.02	0.17	–0.01	–0.14	0.89
Identity, W0	0.15	0.11	0.11	1.34	0.18
Habit strength, W0	–0.05	0.13	–0.03	–0.41	0.68
**Predicting objective calcium supplementation in week 1**					
(Constant)	2.02	0.85		2.36	0.02
Intentions-efficacy, W0	0.60	0.12	0.37	5.05	0.000
Intrinsic motivation, W0	–0.03	0.15	–0.02	–0.19	0.85
Identity, W0	0.09	0.10	0.08	0.95	0.34
Habit strength, W0	–0.02	0.11	–0.02	–0.21	0.84
**Predicting self-reported calcium supplementation in week 4**					
(Constant)	–1.28	0.63		–2.03	0.04
Intentions-efficacy, W3	0.86	0.11	0.55	7.77	0.000
Intrinsic motivation, W3	0.00	0.13	0.00	–0.003	0.997
Identity, W3	0.10	0.11	0.06	0.90	0.37
Habit strength, W3	0.34	0.10	0.24	3.34	0.001
**Predicting objective calcium supplementation in week 4**					
(Constant)	–1.11	0.90		–1.17	0.85
Intentions-efficacy, W3	0.95	0.16	0.54	6.05	0.000
Intrinsic motivation, W3	0.18	0.16	0.11	1.14	0.26
Identity, W3	–0.06	0.14	–0.04	–0.42	0.67
Habit strength, W3	–0.03	0.13	0.02	0.23	0.82

Regression analyses control for intervention group assignment. Results do not meaningfully differ when intervention condition is controlled for or not.

Regarding adherence in later weeks of the study: For week 3 outcomes, intentions-efficacy, intrinsic motivation, and habit strength again predicted calcium supplementation adherence, both self-reported and objectively measured, in bivariate analyses. In simultaneous regression of self-reported adherence, only intentions-efficacy remained a significant predictor; however, for objectively measured adherence, intentions-efficacy and habit strength remained significant predictors. Therefore, there was partial support of the hypothesis that habit strength would become more predictive of behavior in later weeks of the study. However, for Week 4 outcomes, the reverse was true: intentions-efficacy is the only factor that remains a significant predictor in regression analysis of objectively measured adherence, but intentions-efficacy and habit strength remained significant predictors in regression analysis of self-reported frequency. Therefore, overall, we did not find support for the hypothesis that, for calcium supplementation, habit strength would take over as a predictor of behavioral frequency in later weeks, from intentions and self-efficacy, which was expected to predict behavioral frequency initially.

**Hypothesis 6 (behavioral intentions, self-efficacy, and intrinsic motivation will predict exercise frequency and step-counts in initial weeks, whereas habit strength, intrinsic motivation, and exercise identity will predict exercise frequency and step-counts in later weeks).** Regarding initial exercise frequency: Baseline levels of intentions-efficacy predicted self-reported exercise frequency in week 1, in both bivariate and regression analyses. Intrinsic motivation was the only bivariate predictor of Fitbit steps per day in week 1, and no predictors were significant in regression analysis of Fitbit steps. These regression results were the same, with week 1 factors predicting week 2 exercise outcomes—only intentions-efficacy predicted self-reported exercise frequency. In bivariate analyses, however, intentions-efficacy, intrinsic motivation, and habit strength predicted both self-reported and objectively measured exercise. These bivariate analyses partially support the hypothesis that intentions-efficacy and intrinsic motivation would predict exercise frequency in initial weeks. Table 1B shows correlational analyses, and Table 2B shows regression analyses (for weeks 1 and 4 outcomes), for exercise and step-count variables.

**TABLE 1B:** Correlation results for exercise variables.

	Week 1		Week 2		Week 3		Week 4	
				
	Self-report	Objective	Self-report	Objective	Self-report	Objective	Self-report	Objective
1. Intentions-efficacy	0.230**	0.103	0.524**	0.202*	0.499**	0.132	0.596**	0.043
2. Intrinsic motivation	0.095	0.196*	0.242**	0.214*	0.317**	0.131	0.413**	0.201*
3. Identity	0.087	0.117	0.135	0.277**	0.231**	0.177*	0.383**	0.219*
4. Habit strength	0.042	0.159	0.195*	0.221*	0.332**	0.175	0.459**	0.131

The predictors used to calculate each correlation were measured at the preceding timepoint to the outcome. Therefore, for W1 (week 1) Self-Report and Objective outcomes (exercise frequency and step counts), the predictors were measured at baseline (W0). For W2 outcomes, predictors were measured at W1, and so forth.

*P < 0.05 and **P < 0.01.

**TABLE 2B:** Regression results for exercise variables (weeks 1 and 4 outcomes only, for comparison and space reasons).

	B	SE	Beta	T	Sig.
**Predicting self-reported exercise frequency in week 1**					
(Constant)	0.25	1.13		0.22	0.83
Intentions-Efficacy, W0	0.49	0.17	0.23	2.95	0.004
Intrinsic Motivation, W0	0.02	0.15	0.01	0.15	0.88
Identity, W0	0.07	0.17	0.04	0.40	0.69
Habit Strength, W0	0.07	0.13	0.05	0.53	0.60
**Predicting objective physical activity in week 1**					
(Constant)	4404.60	1705.87		2.58	0.01
Intentions-Efficacy, W0	264.34	252.37	0.09	1.05	0.30
Intrinsic Motivation, W0	442.63	256.10	0.18	1.73	0.09
Identity, W0	–143.92	285.32	–0.05	–0.50	0.62
Habit Strength, W0	308.90	206.96	0.14	1.49	0.14
**Predicting self-reported exercise frequency in week 4**					
(Constant)	–0.55	0.48		–1.15	0.25
Intentions-Efficacy, W3	0.57	0.08	0.50	6.96	0.000
Intrinsic Motivation, W3	–0.06	0.16	–0.03	–0.36	0.72
Identity, W3	–0.03	0.12	–0.02	–0.23	0.82
Habit Strength, W3	0.50	0.14	0.30	3.49	0.001
**Predicting objective physical activity in week 4**					
(Constant)	5338.93	1515.74		3.52	0.001
Intentions-Efficacy, W3	–233.66	234.76	–0.11	–1.00	0.32
Intrinsic Motivation, W3	465.61	454.51	0.14	1.021	0.31
Identity, W3	587.29	312.13	0.22	1.88	0.06
Habit Strength, W3	–62.63	387.28	–0.02	–0.16	0.87

Regression analyses control for intervention group assignment. Results do not meaningfully differ when intervention condition is controlled for or not.

Regarding exercise frequency in later weeks of the study: There was partial support for the hypothesis, in that intrinsic motivation, identity, and habit strength (measured in weeks 2 and 3, respectively) were significant bivariate predictors of self-reported exercise frequency in Weeks 3 and 4 (respectively). Counter to expectations, intentions-efficacy remained a significant predictor of self-reported exercise frequency in both weeks 3 and 4. In regression analysis of self-reported exercise frequency in weeks 3 and 4, intentions-efficacy and habit strength were significant predictors. None of the factors were significant predictors in regression analysis of Fitbit activity, and only identity (in week 3) and identity and intrinsic motivation (in week 4) were significant predictors of Fitbit activity in bivariate analyses.

Overall, these results indicate that intentions and self-efficacy remain important for exercise engagement through 1-month post-initiation, and that habit strength, identity, and intrinsic motivation may become more important for exercise frequency over time.

## Discussion

Overall, the results were partly in-line with expectations, in that individuals’ intentions and self-efficacy predicted initial behavioral engagement for both calcium supplementation and exercise, and habit strength increased for both behaviors and became a significant predictor of behavioral frequency in later weeks of the study. However, there were some mixed and unexpected findings that warrant discussion. First, intentions and self-efficacy went starkly down at the end of the study, most likely because participants were only planning to engage in the behaviors for the duration of the study. This speaks to the difficulty in changing behavior when motivation for change is extrinsic (Deci and Ryan, 2000; Ryan and Deci, 2000). The education provided to participants on the importance of getting regular calcium and exercise may not have been enough to persuade the participants to continue with the behaviors after the end of the study. It is unusual for intentions and self-efficacy to be combined. The statistical similarity of the variables to each other and to the outcomes in this study is likely due, at least partially, to the short and similar measures of these constructs.

Second, intrinsic motivation increased (and then decreased) for calcium consumption, which was not expected, since it was not thought that taking calcium supplements could be enjoyable (as might be the case if the calcium supplements were in candy form). However intrinsic motivation items do capture “pleasure and satisfaction” from taking calcium supplements; therefore, participants may have felt a sense of satisfaction in taking their calcium supplements, which may have driven the observed effects. Indeed, medication adherence literature has focused on autonomous motivation (vs. merely intrinsic motivation, or enjoyment) in predicting adherence (e.g., Williams et al., 1998; Kennedy et al., 2004).

Third, habit strength increased for both behaviors, as expected, but it was not expected that habit strength would decrease at the final time-point. A recent study found that some individuals showed a similar “discontinuous habit formation” trajectory, for a nutrition-related behavior (Keller et al., 2021). The dip observed in habit strength and other variables in the present study may be due to the sample and method of compensation (students who were compensated with course credit)—issues of participant motivation; that is, participants’ motivation may have been sufficiently low that they were not invested in the behavior enough to form a habit and to want to continue the behavior after the end of the study. This raises interesting questions about the need for intrinsic or autonomous motivation for forming even relatively simple health-related habits, not just for complex behaviors, such as exercise.

Fourth, counter to expectations, identity with calcium supplementation was higher than for exercise identity throughout, which may be due to a lack of direct comparability between behaviors on identity items. That is, the items differed in ways that may make them inappropriate for direct comparison (i.e., “I see myself as someone who regularly takes calcium” vs. “I see myself as someone who regularly exercises”). Further, we also found that identity did not predict exercise frequency in later weeks, in multivariate analyses. This does not fit with extensive literature showing that exercise identity is associated with exercise frequency (Anderson and Cychosz, 1994; Caldwell et al., 2018; Verplanken and Sui, 2019; Rhodes et al., 2021). Behavioral identity may take much longer to develop than the time observed in this study. Future research should evaluate the mechanisms that may lead to behavioral identity formation (e.g., goal attainment, peer group changes, social identities; Stevens et al., 2017).

Other factors that warrant discussion include the fact that there were mixed results between self-reported and objectively measured behavioral outcomes, for some of the factors. These differences may have been due to common method variance between the self-reported outcome with the self-reported predictive factors. Most of the disagreement was between Fitbit-measured activity and self-reported exercise frequency. These discrepancies are likely due to the fact that the Fitbit-measured variable was not exercise frequency but overall physical activity. Although we would expect greater exercise frequency to manifest in greater activity overall, and therefore concordance between self-reports and Fitbit data, this is not necessarily the case, since some individuals are active throughout the day but never engage in leisure-time purposeful physical activity (“exercise sessions”). Exercise and physical activity are considered distinct constructs with potentially different predictors (Dasso, 2019).

Results also differed between bivariate and multivariate analyses for some of the hypothesis tests. Attenuation of bivariate relationships in multivariate analyses is likely due to the theoretical overlap between factors (intrinsic motivation and habit, intrinsic motivation and intentions, etc.). Future research should evaluate the relative importance of these factors (and whether each construct is a necessary but not sufficient condition) for behavior change and maintenance (Rothman, 2004).

Another limitation is that there were some missing data points, although, the missing data in this dataset are not due to typical attrition seen in other health-behavior intervention trials. The mode of compensation could account for this—the participants were compensated with course credit upon completion of the study and going to a final in-person session to turn in their devices and answer the final survey questions. A final limitation is that the current study compared one simple behavior to one complex behavior, and there may have been other differences between these behaviors that could have affected the results, beyond their relative complexity. The exercise task of going for a brisk walk may not have been particularly difficult or complex for individuals, as well, since the participants were students on a college campus and likely walk a substantial amount incidentally. Future research could compare multiple types of simple and complex behaviors to each other.

The current findings contribute to theories of behavior change and maintenance, in that they suggest the transition to maintenance action control may take longer than a month for both simple and complex health behaviors. This corroborates extant literature that has found habits take a range of times, but with most estimates longer than 4 weeks (Lally et al., 2010; Keller et al., 2021). Further, although the M-PAC (Rhodes et al., 2021) was designed to explain physical activity behavior, the results suggest that identity, intrinsic motivation, and habit strength may be important for simple behaviors and not just for exercise-related behavior. How the MPAC might apply to other health behaviors, whether simple or complex, may also depend on whether the behavior is an action or inaction, intended or counter-intentional behavior, which future research can evaluate. Overall, the current findings suggest that differences in initiation and maintenance between behaviors of differing complexity may not be as stark as theorized (as in Phillips and Mullan, 2022). However, much longer follow-up times are required to evaluate maintenance factors. Lastly, the current findings are too preliminary to suggest specific intervention tactics. For example, even if intentions become less important and habit strength increases as behavior is repeated over time, this does not mean that interventions need focus on intentions and then switch to habit formation strategies. Indeed, habit formation, although a maintenance mechanism, might best be started at behavioral initiation (Lally and Gardner, 2013).

## Data availability statement

The raw data supporting the conclusions of this article will be made available by the authors, without undue reservation.

## Ethics statement

The studies involving human participants were reviewed and approved by Iowa State University IRB. The patients/participants provided their written informed consent to participate in this study.

## Author contributions

LP conceived and designed the study, helped the collect data and supervised data collection, cleaned and analyzed the data, and wrote the manuscript. KM helped the collect data and supervised data collection, provided critical feedback on the current study analyses and manuscript, and edited the manuscript. All authors contributed to the article and approved the submitted version.

## Footnotes

1 N.B. The present study focuses on health promotive behaviors. The role of rewards in habit development and cessation may differ for health risky behaviors, in that health risky behaviors are thought to be inherently rewarding (e.g., addictive) for those who engage in them—e.g., consuming sugary beverages and smoking cigarettes have naturally rewarding biological mechanisms that make them easier to become habitual or addictive.

2 Informed consent did not include consent to publicly share individual-level data. Therefore, we will privately provide access to the original data, codebook, and analysis syntax to any researcher wishing to verify the data presented in this paper. A limitation of this study is that it was not pre-registered, since the study was designed and data collection begun before our lab made it a regular practice to pre-register studies in a publicly accessible repository. However, we aim for transparency by following a multiverse approach to data analysis, which evaluates the robustness of the results. And we encourage interested parties to request the data.
