# Corrigendum: Evaluating behavior change factors over time for a simple vs. complex health behavior

**DOI:** 10.3389/fpsyg.2022.1055474

**Published:** 2022-12-08

**Authors:** L. Alison Phillips, Kimberly R. More

**Affiliations:** ^1^Department of Psychology, Iowa State University, Ames, IA, United States; ^2^Department of Psychology, School of Humanities, Social Sciences and Law, University of Dundee, Dundee, United Kingdom

**Keywords:** behavioral maintenance, theories of behavior change, exercise, adherence, habit formation

In the published article, there was an error. The lead author, LP, discovered that a subset of participants had two survey items within two of the variables of interest switched (and only in a subset of those participants' timepoints). Therefore, in converting the raw data (downloaded from Qualtrics) to SPSS and prior to forming the variables, some participants in the calcium supplementation group had two survey items mis-labeled, from the weekly but not baseline or final follow-up surveys. This affected the final variables used in some of the analyses in the original article. All participants, timepoints, and variables were checked upon detecting this error.

Due to this error, an amendment has been made to the values in Table 1A, Table 2A, and Figures 1B and 1C. The corrected tables and figures appear below.

**Table 1A:** Correlation results for calcium supplementation variables.

	**Week 1**		**Week 2**		**Week 3**		**Week 4**	
	**Self-report**	**Objective**	**Self-report**	**Objective**	**Self-report**	**Objective**	**Self-report**	**Objective**
1. Intentions-efficacy	0.363^**^	0.370^**^	0.492^**^	0.472^**^	0.487^**^	0.438^**^	0.675^**^	0.481^**^
2. Intrinsic motivation	0.017	0.013	0.282^**^	0.269^**^	0.229^**^	0.190^*^	0.359^**^	0.241^**^
3. Identity	0.099	0.055	0.085	0.066	0.096	0.122	0.341^**^	0.108
4. Habit strength	0.012	0.021	0.277^**^	0.252^**^	0.298^**^	0.358^**^	0.561^**^	0.296^**^

The predictors used to calculate each correlation were measured at the preceding timepoint to the outcome. Therefore, for W1 (week 1) self-report and objective outcomes (calcium supplementation frequency), the predictors were measured at baseline (W0). For W2 outcomes, predictors were measured at W1, and so forth.

* P < 0.05 and

** P < 0.01.

**Table 2A:** Regression results for calcium supplementation variables (weeks 1 and 4 outcomes only, for comparison and space reasons).

	**B**	**SE**	**Beta**	**T**	**Sig**.
**Predicting self-reported calcium supplementation in week 1**					
(Constant)	1.31	0.99		1.32	0.19
Intentions-efficacy, W0	0.66	0.14	0.37	4.84	0.000
Intrinsic motivation, W0	−0.02	0.17	−0.01	−0.14	0.89
Identity, W0	0.15	0.11	0.11	1.34	0.18
Habit strength, W0	−0.05	0.13	−0.03	−0.41	0.68
**Predicting objective calcium supplementation in week 1**					
(Constant)	2.02	0.85		2.36	0.02
Intentions-efficacy, W0	0.60	0.12	0.37	5.05	0.000
Intrinsic motivation, W0	−0.03	0.15	−0.02	−0.19	0.85
Identity, W0	0.09	0.10	0.08	0.95	0.34
Habit strength, W0	−0.02	0.11	−0.02	−0.21	0.84
**Predicting self-reported calcium supplementation in week 4**					
(Constant)	−1.28	0.63		−2.03	0.04
Intentions-efficacy, W3	0.86	0.11	0.55	7.77	0.000
Intrinsic motivation, W3	0.00	0.13	0.00	−0.003	0.997
Identity, W3	0.10	0.11	0.06	0.90	0.37
Habit strength, W3	0.34	0.10	0.24	3.34	0.001
**Predicting objective calcium supplementation in week 4**					
(Constant)	−1.11	0.90		−1.17	0.85
Intentions-efficacy, W3	0.95	0.16	0.54	6.05	0.000
Intrinsic motivation, W3	0.18	0.16	0.11	1.14	0.26
Identity, W3	−0.06	0.14	−0.04	−0.42	0.67
Habit strength, W3	−0.03	0.13	0.02	0.23	0.82

Regression analyses control for intervention group assignment. Results do not differ meaningfully when intervention condition is controlled for or not.

**Figure 1b:**
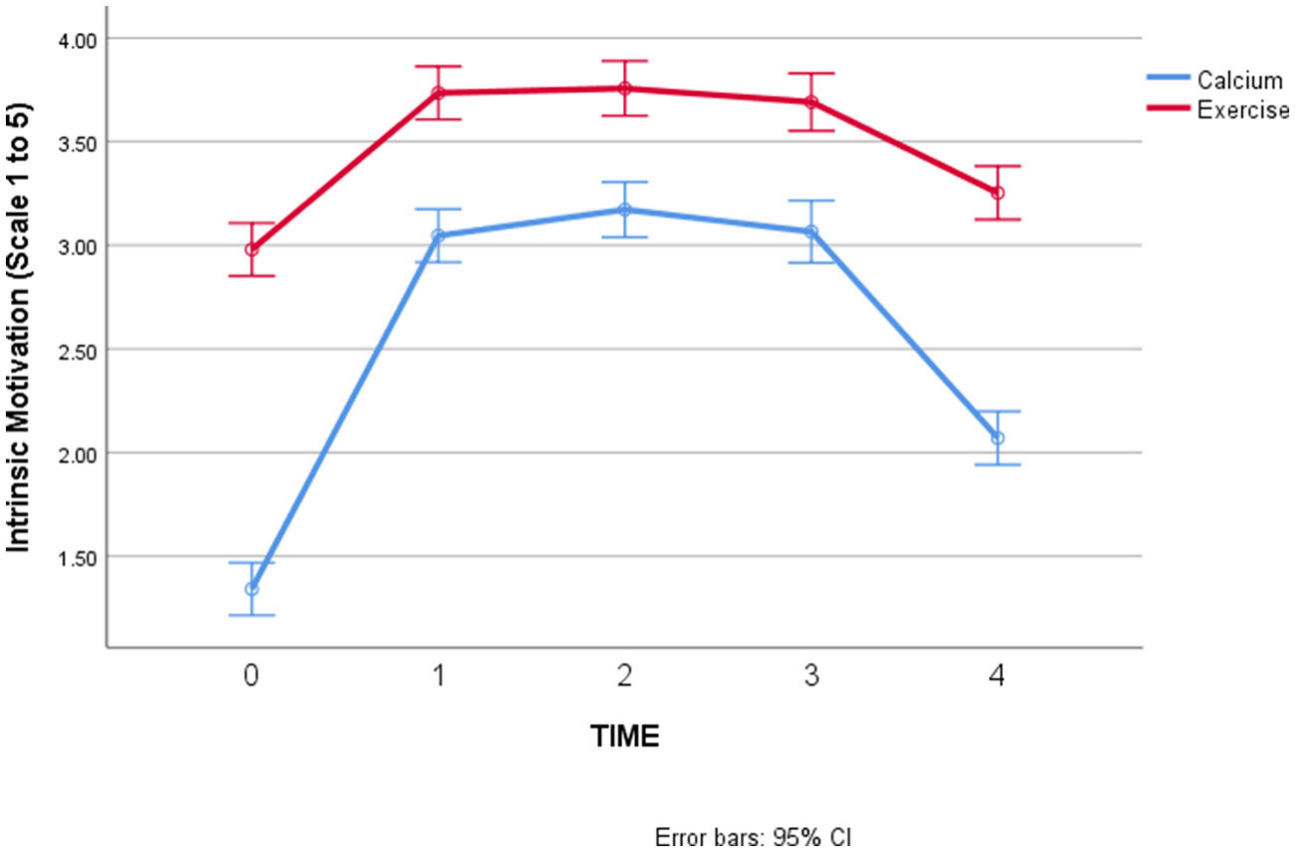
Changes in intrinsic motivation over time, by behavior.

**Figure 1c:**
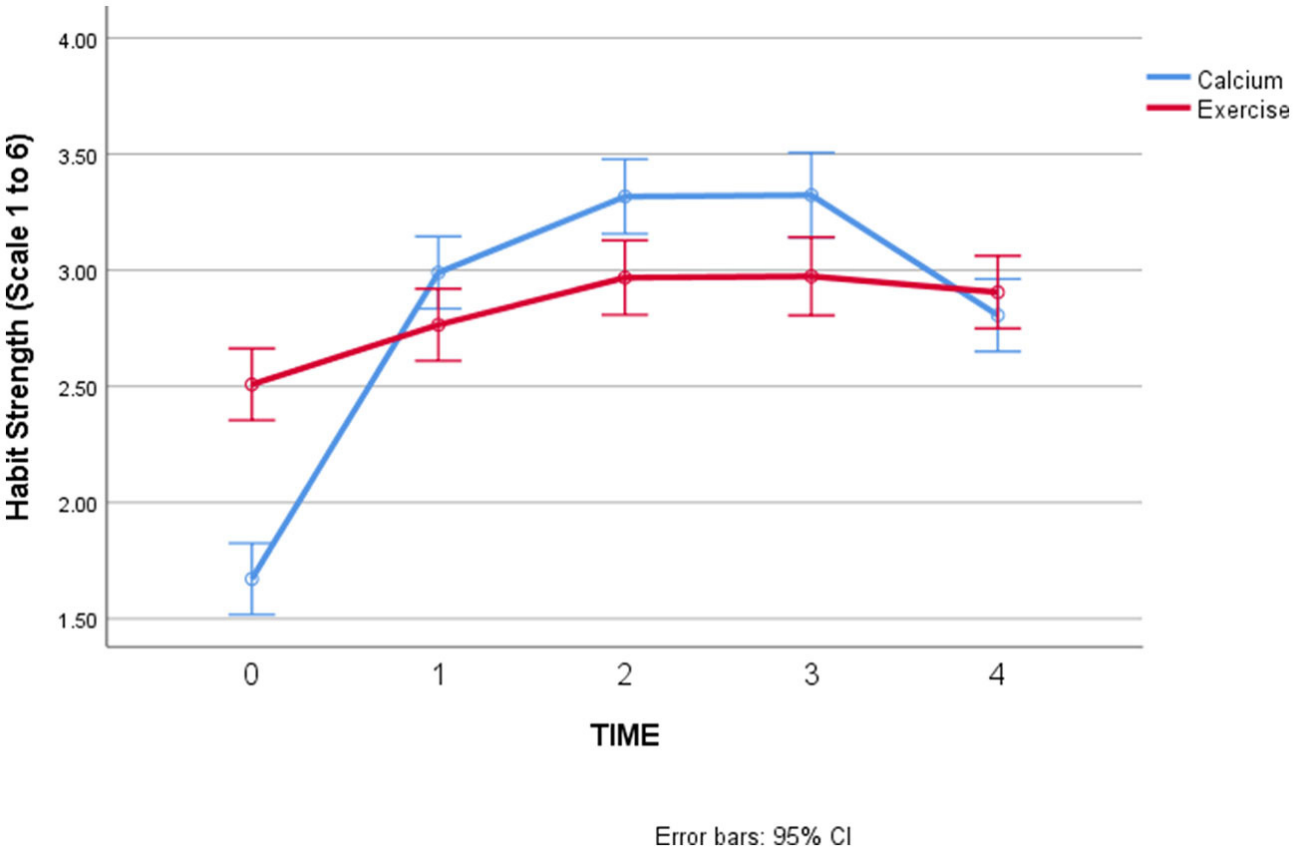
Changes in habit strength over time, by behavior.

In addition, a correction has been made to Results, Paragraph 2. “*F* = 44.24” should be “*F* = 23.57.” The corrected paragraph appears below:

**Hypothesis 2 (intrinsic motivation will start and remain low for calcium supplementation but will increase over time for exercise)**. There were significant main effects of Time and Behavior in predicting intrinsic motivation over time, but these were qualified by a significant interaction between Time and Behavior (*F* = 23.57, *p* < 0.001). Figure 1B shows the results for intrinsic motivation, with 95% confidence intervals on the mean levels of intrinsic motivation for each behavior at each timepoint: As hypothesized, intrinsic motivation was higher for exercise than calcium at the start and throughout the study. However, intrinsic motivation did increase (and then decrease) for calcium consumption, which was not expected.

A correction has also been made to Results, Paragraph 3. “*F* = 12.11” should be “*F* = 19.54” and “a slight decrease at the final timepoint for both calcium supplementation and exercise” should be “a slight decrease at the final timepoint for calcium supplementation.” The corrected paragraph appears below:

**Hypothesis 3 (habit strength will start low and increase over time for both behaviors)**. There were significant main effects of Time and Behavior in predicting habit strength over time, which were qualified by a significant interaction between Time and Behavior (*F* = 19.54, *p* < 0.001). As seen in Figure 1C, there was an overall increase in habit strength, with a slight decrease at the final timepoint for calcium supplementation. We note that neither behavior shows an average level of habit strength = 4, which is a rough level associated with “having a habit,” since individuals have to agree on average with the statements that their engagement in the behavior is “automatic,” or habitual, to have a score of 4.

A correction has also been made to Results, Paragraph 6. The discussion of Week 4 outcomes has been amended. The corrected paragraph appears below:

Regarding adherence in later weeks of the study: For week 3 outcomes, intentions-efficacy, intrinsic motivation, and habit strength again predicted calcium supplementation adherence, both self-reported and objectively measured, in bivariate analyses. In simultaneous regression of self-reported adherence, only intentions-efficacy remained a significant predictor; however, for objectively measured adherence, intentions-efficacy and habit strength remained significant predictors. Therefore, there was partial support of the hypothesis that habit strength would become more predictive of behavior in later weeks of the study. However, for Week 4 outcomes, the reverse was true: intentions-efficacy is the only factor that remains a significant predictor in regression analysis of objectively measured adherence, but intentions-efficacy and habit strength remained significant predictors in regression analysis of self-reported frequency. Therefore, overall, we did not find support for the hypothesis that, for calcium supplementation, habit strength would take over as a predictor of behavioral frequency in later weeks, from intentions and self-efficacy, which was expected to predict behavioral frequency initially.

The authors apologize for these errors and state that this does not change the scientific conclusions of the article in any way. The original article has been updated.

## Publisher's note

All claims expressed in this article are solely those of the authors and do not necessarily represent those of their affiliated organizations, or those of the publisher, the editors and the reviewers. Any product that may be evaluated in this article, or claim that may be made by its manufacturer, is not guaranteed or endorsed by the publisher.

